# The Crucial Role of Laboratory Medicine in Addressing Future Public Health Infectious Threats: Insights Gained from the COVID-19 Pandemic

**DOI:** 10.3390/diagnostics15030323

**Published:** 2025-01-30

**Authors:** Giuseppe Lippi, Brandon M. Henry, Camilla Mattiuzzi

**Affiliations:** 1 Section of Clinical Biochemistry, University of Verona, Ospedale Policlinico GB Rossi, 37134 Verona, Italy; giuseppe.lippi@univr.it; 2 Clinical Laboratory, Division of Nephrology & Hypertension, Cincinnati Children’s Hospital Medical Center, Cincinnati, OH 45229, USA; 3 Evidence-Based Medicine and Laboratory Research Collective, San Antonio, TX 78201, USA; 4 Medical Direction, Rovereto Hospital, Provincial Agency for Social and Sanitary Services (APSS), 38068 Rovereto, Italy

**Keywords:** laboratory medicine, COVID-19, SARS-CoV-2, lessons

## Abstract

Laboratory testing has played a pivotal role throughout the coronavirus disease 2019 (COVID-19) pandemic, exemplifying the importance of in vitro diagnostics in addressing public health threats posed by outbreaks of infectious diseases. This article aims to present key insights from our expertise, derived from evidence gathered during the COVID-19 pandemic, to inform strategies for managing future infectious challenges. Current scientific evidence underscores that patient sample testing not only allows to diagnose an acute severe acute respiratory syndrome coronavirus 2 (SARS-CoV-2) infection, but also supports outbreak prediction, improved control measures, anticipation of pressure on the healthcare system, mitigation of adverse clinical outcomes, and early detection of emerging SARS-CoV-2 variants. Additionally, wastewater monitoring has emerged as a powerful tool for forecasting disease burden, including both prevalence and severity. Collectively, these findings underscore the value of diagnostic testing and wastewater surveillance in guiding healthcare planning and optimizing resource allocation during the COVID-19 pandemic, offering a valid framework to be applied to future public health threats, especially to any potential outbreak of “Disease X” that may emerge in the future.

## 1. Introduction

The coronavirus disease 2019 (COVID-19) pandemic was a globally disruptive event of unprecedented scale, profoundly affecting all domains of modern life. Healthcare systems worldwide have experienced extraordinary strain, with hospitals and other medical facilities overwhelmed by surges in critically ill patients [1]. Intensive care units (ICUs) rapidly reached full capacity, necessitating ethically and logistically challenging decisions regarding the allocation of limited human and technical resources [1]. Consequently, routine medical care was significantly disrupted, including delays or cancellations of screenings [2], elective procedures, and non-urgent medical treatments [3,4]. Substantial delays in emergency healthcare services also compounded these challenges [5], collectively contributing to widespread downstream health consequences on a global scale.

Recent history has underscored the pivotal role played by laboratory medicine in responding to the COVID-19 pandemic, despite the significant challenges faced by this field [6]. Diagnostic laboratories were overwhelmed by the unprecedented demand for severe acute respiratory syndrome coronavirus 2 (SARS-CoV-2) testing, necessitating rapid scaling of capacity, infrastructure, instrumentation, and workforce. Evidence highlights that clinical laboratories were largely unprepared for this unexpected challenge, as demonstrated by a comprehensive European survey [7]. Specifically, nearly 70% of respondents reported shortages of non-COVID reagents, while over 78% faced shortages of COVID-specific reagents. Moreover, 61% admitted that they were unable to complete all the SARS-CoV-2 tests required during the early stages of the pandemic. Alarmingly, 72.3% of laboratory professionals reported experiencing varying degrees of exhaustion or burnout during this period. These situations should be regarded as critical lessons for the future, enabling more proactive preparedness to address upcoming infectious challenges. An important premise that needs to be addressed is that the COVID-19 pandemic evidenced large disparities in healthcare systems worldwide, especially between the public and private sectors. This heterogeneity has had a considerable impact on access to essential services, including molecular testing, which are critical for accurately assessing the extent of viral spread and its implications for public health. Notably, these disparities have also encompassed vaccine distribution, health education, and the capacity for timely intervention, further complicating efforts to manage the pandemic. Strengthening global health equity through improved resource allocation, collaboration, and investments in robust healthcare services will hence be essential to mitigate the consequences of current and future health crises.

## 2. The Critical Impact of Patient Sample Testing During the COVID-19 Pandemic

Despite numerous challenges, the COVID-19 pandemic underscored the vital role of laboratory medicine and patient sample testing in managing public health crises, including both COVID-19 and other global emergencies. Beyond its essential role in diagnosing acute SARS-CoV-2 infections [8], this section explores key aspects of patient sample testing that emerged throughout the course of the ongoing pandemic, primarily derived from our expertise, to highlight important lessons that can guide strategies for managing future infectious challenges (Table 1). To support the discussion, relevant bibliographic references were identified through systematic searches of scientific databases.

### 2.1. Prediction and Better Control of SARS-CoV-2 Outbreaks

Integrating laboratory testing into infectious disease surveillance programs can significantly enhance the prediction, prevention, and management of local outbreaks. Accurate and timely test results—whether positive, negative, or inclusive of viral load—enable the rapid identification, isolation, and clinical management of infectious individuals, thereby reducing the risk of transmission both within households and in the broader community. Additionally, incorporating laboratory data into predictive modeling improves outbreak forecasting by identifying transmission patterns and hotspots [8].

These key aspects have been confirmed by a number of studies. Zhu et al. developed a logistic model based on SARS-CoV-2 testing, the date of the infection, generation time distributions, and locations to estimate the transmission distance of infections [9]. It was found that SARS-CoV-2 population screening by means of molecular testing within five days from identification of the first positive case would permit confining the outbreak within a limit of 2.2 km, thus effectively and rapidly stopping the outbreak from becoming larger. In another study, Grassly et al. explored the effectiveness of a weekly screen of high-risk groups irrespective of symptoms by means of molecular biology techniques [10] and estimated that SARS-CoV-2 transmission could be reduced by over 20%, thus contributing to the early stop of local outbreaks by timely isolation of positive cases. These important aspects were summarized in the review article by Mercer and Salit [11], who concluded that most population-scale testing programs resulted in a significant mitigation of SARS-CoV-2 transmission and reduction of reproductive numbers, which ultimately reflects the burden of viral transmission within a specific population.

Early identification by timely SARS-CoV-2 testing also provides incremental benefits in circumscribed environments populated by subjects at higher risk of unfavorable clinical courses, such as residents of long-term care homes. In the study of Brown et al. [12], early outbreak detection (i.e., within 2 infectious resident-days) was associated with a much lower rate of secondary infections compared to late (i.e., ≥3 infectious resident-days) outbreak detection (3.3 vs. 10.3%; *p* < 0.001).

### 2.2. Prediction of COVID-19-Related Healthcare Pressure

Reliable evidence demonstrates that the cumulative number of SARS-CoV-2 tests and associated positivity rates are valuable predictors of healthcare system pressure. A 2021 study by Lippi and Mattiuzzi in Italy [13] found significant correlations between the number of positive SARS-CoV-2 tests or test positivity rate and key healthcare metrics. Specifically, positive test counts correlated with hospital admissions after 20–23 days (r = 0.74) and ICU admissions after 16–21 days (r = 0.61), while test positivity rates showed even stronger correlations for hospital admissions (r = 0.86), for ICU admissions (r = 0.92), and for cumulative deaths (r = 0.92) after 17–20 days. Similarly, López-Izquierdo et al. conducted a study in Spain [14] that found a significant association (*p* < 0.001) between daily positive test percentages and hospital admissions within the subsequent 10 days. In the US, Scobie et al. [15] used nationwide COVID-19 surveillance data to monitor healthcare pressure, showing that positive test results from CELR (COVID-19 Electronic Laboratory Reporting) and NREVSS (National Respiratory and Enteric Viruses Surveillance System) significantly anticipated hospital admissions by four days (r = 0.48 and r = 0.43, respectively).

More recently, in 2023, Klein et al. [16] developed a multi-step, recursive predictive model incorporating statewide test positivity data with aggregated mobile device information to improve predictions of hospital admissions. This enhanced model extended prediction efficiency by up to three weeks (specific correlation metrics were not reported). Additionally, Hatfield et al. [17] analyzed SARS-CoV-2 testing data from the PINC (Performance, Innovation, Network, and Care) Healthcare Database between July 2020 and June 2022. Their cohort study revealed that each 10% increase in the community-based SARS-CoV-2 test positivity rate was associated with a nearly threefold increase in hospital-onset infection rates (rate ratio, 2.78; 95% CI, 2.52–3.07).

In summary, studies from various countries consistently highlight a significant association between SARS-CoV-2 test positivity rates and subsequent burdens on healthcare facilities. This relationship is attributable to the fact that higher positivity rates reflect increased community transmission, which translates into the probability of more severe COVID-19 cases and higher strain on hospital resources, including ICU capacity.

### 2.3. Impact on Hospital or Intensive Care Unit Admissions Averted and Lives Saved

Regarding the relationship between SARS-CoV-2 testing and COVID-19 clinical outcomes, several studies have provided compelling evidence. Kannoth et al. [18] analyzed data from the Our World in Data database, which included laboratory tests and mortality records from 27 countries between 31 December 2019, and 30 September 2020. Using Cox proportional hazards regression, they identified a significant inverse association (r = −0.59; *p* < 0.001) between early COVID-19 testing capacity and subsequent mortality, even after adjusting for confounders such as age, population density, hospital bed capacity, nonpharmaceutical interventions, and gross domestic product. Similarly, Wei et al. [19] demonstrated a strong inverse correlation (r = −0.79; *p* < 0.001) between SARS-CoV-2 testing coverage and mortality rates across 36 OECD (Organization for Economic Development) countries and Taiwan, with higher testing coverage linked to lower country-level mortality.

In the US, Santos et al. [20] conducted a descriptive analysis of COVID-19 outcomes from January 1, 2020, to December 31, 2022. They developed a predictive model to evaluate the impact of laboratory testing under four scenarios: (a) a 20% reduction in testing availability, (b) a six-month delay in scaling up testing, (c) historical testing trends, and (d) a hypothetical 2.8-fold increase in testing capacity. The findings revealed that both the third and fourth scenarios (current test availability and increased testing) were associated with approximately 1.4 million lives saved and nearly 7 million hospitalizations averted. Additional insights were provided by Zhang et al. [21], who demonstrated that the introduction of widespread community testing using rapid diagnostic tests for SARS-CoV-2 antigens (RDT-Ag) was associated with a 43% reduction in future COVID-19-related hospital admissions (95% CI, 29–57%).

Regarding ICU admissions, a French group conducted a retrospective study to assess the relationship between SARS-CoV-2 test positivity rates and ICU admissions [22]. Their results showed that the positive test rate strongly predicted total ICU occupancy with a 15-day lead time (r = 0.97) and new ICU admissions with a 4-day lead time (r = 0.92).

Collectively, these findings highlight the critical role of widespread and timely SARS-CoV-2 testing (and official recording) in reducing mortality, mitigating hospital and ICU admissions, and ultimately improving COVID-19 clinical outcomes.

### 2.4. Early Identification of Emergence of New SARS-CoV-2 Variants

Timely detection of emerging SARS-CoV-2 variants is crucial for rapid risk assessment and coordination of public health actions, such as preparing healthcare facilities for admission of new cases and updating vaccine formulations. Increased detection of new positive cases and higher positivity rates may signal the emergence of new variants, prompting the need for genomic surveillance.

Han et al. conducted a simulation of COVID-19 epidemics in low- and middle-income countries to explore how testing rates influence the identification of new variants [23]. Their findings revealed that low testing rates were associated with significant delays—ranging from weeks to months—before new variants could be detected. A cost-effective approach for timely detection of new SARS-CoV-2 variants was identified as testing nearly 100 samples per 100,000 inhabitants per day.

In another simulation study, Lippi et al. developed a simple epidemiological model to predict the emergence of new SARS-CoV-2 variants [24]. One key component of this model, in addition to the date of emergence and cumulative COVID-19 vaccination doses, was the cumulative number of new positive SARS-CoV-2 tests. A strong inverse correlation (r = 0.96; *p* = 0.001) was found between the number of new positive cases and the neutralization potential of the recently emerged SARS-CoV-2 sublineages. These findings underscore the close relationship between the number of new positive SARS-CoV-2 tests and the emergence of new sublineages, as novel variants often evade immunity developed against previous strains, leading to a surge in infections. This was observed, for example, during the rise in COVID-19 cases, corresponding to the emergence of the Omicron XBB and JN.1 sublineages [25].

Although various factors such as infectivity, immune evasion, and implementation of public health measures influence the total number of new COVID-19 cases, a significant increase in positive SARS-CoV-2 tests may serve as a strong indicator of the potential emergence of a new variant.

## 3. Wastewater Testing

Wastewater testing, which includes wastewater surveillance and wastewater-based epidemiology, involves analyzing sewage to monitor public health [26]. By detecting pathogens, chemicals, drugs, and other substances in community wastewater, this approach provides valuable insights into the health status of populations, without the need for individual testing. Wastewater testing has proven instrumental in tracking various infectious diseases, as recently reviewed by Singh et al. [27]. In this section, we will examine key evidence from wastewater testing in the context of COVID-19 and explore how these insights can inform strategies for addressing future pandemic challenges.

### 3.1. Technical Issues

Before delving into the role of wastewater testing in monitoring COVID-19 and addressing future infectious public health threats, it is important to clarify several technical and methodological considerations that distinguish wastewater testing from traditional patient sample analysis (Table 2) [26,27].

The first challenge is that the sensitivity of the technique can variably depend on equipment and methodology. Since pathogen concentrations in wastewater are significantly lower than in human samples, various concentration methods are employed to isolate and concentrate viral RNA, such as ultrafiltration, precipitation, and adsorption to magnetic beads. However, these methods can introduce biases or inaccuracies, such as viral particle loss or contamination, which may reduce the overall diagnostic sensitivity [28]. Notably, reverse transcription quantitative polymerase chain reaction (RT-qPCR) is generally outperformed by reverse transcription digital PCR (RT-dPCR) in terms of analytical sensitivity, specifically showing a lower limit of detection [28]. Primer design is another crucial factor influencing assay sensitivity. To optimize detection, it is preferable to use highly specific SARS-CoV-2 sequences that minimize cross-reactivity with other genetic material in wastewater. Targeting more conserved regions of the SARS-CoV-2 genome, such as nucleocapsid (*N*) or RNA-dependent RNA polymerase (*RdRp*) genes, enhances specificity and sensitivity [29]. Next-generation sequencing (NGS) also offers the potential to detect complete SARS-CoV-2 genomes in wastewater, which can provide a more detailed understanding of the circulating variants within a population. However, NGS requires additional resources, time, and expertise to be effectively implemented [30].

Additional challenges, as summarized in Table 2, include the complex composition of wastewater samples, which may contain contaminants that interfere with testing or cause sample degradation. Such factors can ultimately reduce the sensitivity and specificity of detection assays. Sampling issues also arise, as individual specimens may not accurately represent the broader composition of wastewater across different geographical locations or timeframes. Variations in wastewater concentration may occur due to factors like heavy rainfall (which can dilute samples) or prolonged droughts (which can concentrate them). Another challenge is the lack of harmonization, as there are no uniform protocols or standardized techniques for sampling, preparing, and analyzing wastewater specimens. This variability makes it difficult to compare results across studies. Establishing a systematic wastewater surveillance program also requires considerable investment in infrastructure, personnel, and equipment for sample collection, transport, and analysis. Additionally, the interpretation of a single sample is limited, as it does not provide a comprehensive picture of community health, such that longitudinal sampling is necessary to obtain more accurate and reliable insights [31].

### 3.2. Practical Advantages

Several studies have explored the role of wastewater monitoring in predicting COVID-19 case surges, hospitalizations, ICU admissions, and deaths, with some key findings summarized below.

Galani et al. assessed SARS-CoV-2 RNA levels in raw wastewater for environmental surveillance in Attica, Greece, over a six-month period [32]. The study revealed that SARS-CoV-2 viral load in wastewater was a strong predictor of COVID-19 cases (r = 0.986), new hospitalizations (r = 0.975), and ICU admissions (r = 0.992) with 5-, 8- and 9-day delays, respectively. Similarly, Hegazi et al. examined SARS-CoV-2 levels in wastewater from Ottawa and Hamilton, Canada [33], and found significant correlations with new COVID-19 diagnoses (ρ = 0.821 and 0.655 for the two sites, respectively; 5 to 10 days’ lag), hospital admissions (ρ = 0.808 and 0.682; 10 to 14 days’ lag), ICU admissions (ρ = 0.708 and 0.352; 10 to 14 days’ lag), and deaths (ρ = 0.730 and 0.574; 19 to 20 days’ lag) before the global vaccination campaigns started. After mass immunization, the associations were even stronger, with higher correlations between wastewater SARS-CoV-2 RNA and clinical outcomes like new SARS-CoV-2 diagnoses (ρ = 0.861 and 0.727; no lag), COVID-19-related hospital admissions (ρ = 0.947 and 0.837; 3–4 days’ lag), ICU admissions (ρ = 0.911 and 0.673; 2–14 days’ lag), and deaths (ρ = 0.905 and 0.663; 9–18 days’ lag) [33].

A large-scale study by Li et al. [34] investigated wastewater-based epidemiology (WBE) to predict new COVID-19 hospitalizations across 159 counties and 45 US states, covering nearly 100 million people. Their model showed high accuracy, with correlation coefficients of 0.82, 0.81, 0.82, and 0.82 with 1-, 2-, 3- and 4-week lead times. Another study by Schenk et al. [35] used data from 189 wastewater treatment plants across 40 US states between 2023 and 2024, finding that SARS-CoV-2 RNA in wastewater predicted COVID-19 hospitalizations, with a median correlation of 0.85, ranging from 2 to 12 days in advance.

In 2024, Rankin et al. [36] developed a multi-city wastewater-based model in the US to predict COVID-19-related hospitalizations. Their findings showed that wastewater SARS-CoV-2 concentrations predicted future hospitalization capacity risk and hospitalization trends, with accuracies of 88.8% and 69.6% after one week, 82.3% and 56.4% after two weeks, and 74.8% and 45.2% after three weeks, respectively. More recently, Kadoya et al. established a state-space model in Japan, encompassing wastewater viral monitoring, which was effective for predicting reported COVID-19 cases and infection numbers with a one-week delay and high accuracy (fold difference value between estimated and real cases always <1) [37]. 

In summary, similar to patient sample testing, wastewater monitoring has become an important tool for tracking the spread of COVID-19. It provides key insights into community infection levels and anticipates future healthcare system pressures. Nonetheless, an important aspect to consider in this context is the pandemic-induced surge of industrial, household, and even infectious waste, which has presented significant environmental challenges. Improper management of this waste may have the potential to generate unforeseen adverse impacts on both human health and the ecosystem, thus highlighting the need for developing and implementing more robust, efficient, and safe waste management strategies during the current pandemic and to prepare for future infectious disease outbreaks.

## 4. Conclusions

A pivotal lesson derived from the COVID-19 pandemic, with broad applicability to future outbreaks or pandemics (e.g., new SARS-CoV-2 variants causing different symptoms, bird flu, mpox, and even Metapneumovirus), is that the first 100 days are critical for implementing scalable disease management and containment mechanisms, which include the development and distribution of accurate, rapid, and efficient diagnostic tests [38,39,40]. The scientific literature reviewed in this article emphasizes that patient delving into molecular surveillance for COVID-19 is highly worthwhile, as it has enabled—and continues to enable—the diagnosis of acute SARS-CoV-2 infections. Moreover, it plays a crucial role in predicting and controlling SARS-CoV-2 outbreaks, anticipating healthcare system strain, reducing unfavorable clinical outcomes, and facilitating early detection of new SARS-CoV-2 variants. In addition to patient sample testing, wastewater monitoring has also proven to be a valuable tool in forecasting disease burden—both in terms of volume and severity. These crucial insights must be applied to future public health threats, particularly to any potential “Disease X” that may emerge in the foreseeable future [41].

## Figures and Tables

**Table 1:** Key aspects of laboratory medicine during the COVID-19 pandemic that may play a role in addressing future public health threats.

Diagnosis of acute SARS-CoV-2 infection Prediction and better control of SARS-CoV-2 outbreaks Prediction of COVID-19-related healthcare pressure Reduction of unfavorable COVID-19 clinical outcomes Early identification of the emergence of new SARS-CoV-2 variants

**Table 2:** Leading problems in wastewater testing for the purpose of addressing future public health threats.

Analytical sensitivity Interference from contaminants Dilution or concentration Sampling problems Standardization issues Resource-demanding Interpretation challenges

## Data Availability

No new data were created or analyzed in this study. Data sharing is not applicable to this article.

## Abbreviations

The following abbreviations are used in this manuscript:

COVID-19	Coronavirus Disease 2019
SARS-CoV-2	Severe Acute Respiratory Syndrome Coronavirus 2
ICU	Intensive Care Unit
CELR	COVID-19 Electronic Laboratory Reporting
NREVSS	National Respiratory and Enteric Viruses Surveillance System
OECD	Organization for Economic Development
RDT-Ag	Antigen Rapid Diagnostic Test
RT-qPCR	Reverse Transcription Quantitative Polymerase Chain Reaction
RT-dPCR	Reverse Transcription Digital Polymerase Chain Reaction
N	Nucleocapsid
RdRp	RNA-Dependent RNA polymerase
NGS	Next-Generation Sequencing
WBE	Wastewater-Based Epidemiology
